# Gallium maltolate causes genomic instability to enhance glioblastoma response to radiation and temozolomide

**DOI:** 10.1093/noajnl/vdag246

**Published:** 2026-09-11

**Authors:** Joan N Whittier, Stephenson B Owusu, John Cooke, Jann N Sarkaria, Michael S Petronek

**Affiliations:** Department of Radiation Oncology, University of Iowa, Iowa City, Iowa; Department of Radiation Oncology, University of Iowa, Iowa City, Iowa; Department of Radiation Oncology, University of Iowa, Iowa City, Iowa; Department of Radiation Oncology, Mayo Clinic, Rochester, Minnesota; Department of Radiation Oncology, University of Iowa, Iowa City, Iowa

**Keywords:** DNA damage, gallium maltolate, glioblastoma, iron metabolism, mitochondria

## Abstract

**Background:**

Glioblastoma (GBM) represents the most common and aggressive central nervous system malignancy where overall survival is a dismal 14.6 months despite an aggressive standard of care treatment regimen that includes maximally safe surgical resection, radiotherapy, and temozolomide. It has recently been established that GBM tumors have increased transferrin receptor expression and iron content which corresponds with worse patient prognosis. This has led to the hypothesis that iron metabolism is a viable therapeutic target. An emergent therapeutic strategy to target GBM iron metabolism is gallium maltolate (GaM), which is an orally bioavailable form of Ga^3+^ that functions as a redox inactive iron mimic to disrupt iron metabolism. As mitochondrial iron metabolism serves as a central connection point between iron and DNA metabolism, the goal of this study was to determine if GaM could disrupt this system.

**Methods:**

The effects of GaM on GBM cells were interrogated using conventional techniques including colony formation assays to assess clonogenicity, alkaline comet assays to evaluate DNA damage induction, and Mito-FerroGreen analysis to evaluate mitochondrial iron content. The effects of GaM on tumor growth in vivo were evaluated using a subcutaneous flank xenograft model system to determine the induction of DNA damage and an orthtopic model system to evaluate the combinatorial effects of GaM, radiation, and temozolomide on murine survival.

**Results:**

This study uncovered that maintenance of high mitochondrial iron content corresponds with increased proliferative capacity in GBM cells. This study also shows that GaM can deplete mitochondrial iron to induce DNA damage both in vitro and in a subcutaneous xenograft model. Furthermore, GaM enhances the efficacy of radiation and temozolomide when delivered concomitant and adjuvantly in an orthotopic GBM model system.

**Conclusion:**

These results suggest that GaM warrants further consideration for use in combination with standard of care GBM therapy.

Key Points

- High mitochondrial iron demand is associated with glioblastoma (GBM) cell proliferation.
- Gallium maltolate induces DNA damage in highly proliferative GBM cells.
- Gallium maltolate enhances the toxicity of radiation and temozolomide in preclinical GBM models.

Importance of the Study

Aggressive glioblastoma (GBM) has been characterized phenotypically with an apparent iron dependency which has suggested that iron modulating therapies would be beneficial. Gallium maltolate (GaM) represents a therapeutic strategy with significant interest in the GBM community which has been shown to cause iron deprivation and inhibit tumor growth. To this point, most studies have focused on the utility of GaM alone. This study reports the ability of GaM to cause deplete mitochondrial iron and induce DNA damage as a novel mechanism of action. Moreover, this study provides first evidence for the potential utility of GaM in combination with standard of care (radiotherapy and temozolomide) where there is enhanced cell killing in vitro and enhanced overall survival in an orthotopic mouse model.

Glioblastoma (GBM) represents the most common and aggressive central nervous system malignancy.^1-3^ The current standard of care (SOC) treatment is maximally safe surgical resection, radiotherapy, and temozolomide; however, this therapeutic regimen only yields a median overall survival of 14.6 months and progression-free survival of 6-7 months. There have been potentially beneficial additions to the standard of care recently using tumor-treating fields or pharmacological ascorbate which improved overall survival to 18.8 and 19.6 months, respectively.^4-9^ However, there is still a need for more efficacious therapies to improve patient outcomes.

An important phenotypic discovery recently demonstrated that GBM have increased transferrin receptor (TfR) expression and high iron content when compared to normal brain tissue.^10^^,^^11^ Moreover, higher TfR and iron content is predictive of worse patient outcomes.^11^ While serving as useful prognostic insights, these discoveries are highly suggestive that the maintenance of high iron content is an important underlying feature to support tumor progression. Yet the mechanistic role of iron promoting tumor progression is still unclear.

Iron plays a critical role in the maintenance of cellular metabolism as it is an important cofactor for a myriad of enzyme-driven homeostatic processes. The mitochondria are of particular interest when considering iron-dependent proliferation, as it houses the machinery required for the synthesis of iron-sulfur clusters which are a direct molecular connection between iron and DNA metabolism.^12^ Following formation, these iron-sulfur clusters are used as cofactors in an array of nuclear DNA metabolic enzymes (eg DNA primase, DNA polymerases, DNA helicases) that are responsible for maintaining genome stability.^13^ Therefore, we hypothesize that targeting mitochondrial iron metabolism can be a useful therapeutic strategy to destabilize the genome and increase cellular sensitivity to radiation and temozolomide.

An emergent strategy for accomplishing this goal is gallium maltolate (GaM). Gallium maltolate is a modernized, orally bioavailable formulation of Ga^3+^. Ga^3+^ can act as an antagonist to divalent metal ions (eg Fe^2+^) which can be taken into cells through TfR-mediated endocytosis and function inside the cell as a redox inactive iron mimic.^14^ The cytotoxicity of Ga^3+^ has been largely owed to its ability to inhibit specific iron-containing proteins such as ribonucleotide reductase and/or complex 1 of the electron transport chain.^15^ Another form of Ga^3+^, Ga(NO_3_)_3_, has recently been shown to cause mitochondrial iron depletion and induce DNA damage, which suggests that the cytotoxic effects of GaM may not be limited to just these specific iron-containing enzymes.^16^ The overall goal of this study was to test the hypothesis that GaM could deplete mitochondrial iron and induce DNA damage, subsequently serving as a strategy to enhance the effectiveness of radiation and temozolomide in GBM.

## Methods

### Cell Culture

All glioma cells (U251, Millipore Sigma; GBM06, and GBM39) were cultured in DMEM-F12 media (15% FBS, 1% penicillin-strep, 1% Na-pyruvate, 1.5% HEPES, 0.1% insulin, and 0.02% fibroblast growth factor) and grown to 70%-80% confluence at 21% O_2_ before experimentation. GBM06 and GBM39 are both isocitrate dehydrogenase wild-type, patient-derived cell lines that were obtained from a primary tumor. All cell lines were authenticated before use.

### Cell Culture Treatments

Cells were treated GaM (Sigma-Aldrich #ADVH9B9B4698) from a 50 mM stock in H_2_O at a standard dosing concentration of 50 µM for 24 h unless noted otherwise. All GaM treatments were done prior to any subsequent treatments. Irradiation was done using an Xstrahl CIX3 cabinet X-ray irradiator (Xstrahl, Inc.). Cells were treated with 2.5 µM temozolomide (Sigma-Aldrich # T2577) for 1 h following irradiation and then removed from the media.

### Clonogenic Survival

For colony formation assays, cells were plated as single cells (500-2000 cells per dish) and allowed to grow for 7-12 days to allow for colony formation. Patient-derived cells were left for ≥21 days to form colonies. Following colony growth, cells were washed, fixed to the dish with 70% EtOH (stored at 4°C), and stained with Coomassie blue. After the completion of treatment, cells were trypsinized, counted, and replated as single cells for colony formation. Colonies with >50 cells were counted. For synergy experiments, cells were initially plated as single cells and left for 24 h to attach to the dish. Cells were then treated with GaM (12.5, 25, 50 µM) for 24 h after which the media was replaced and the cells were irradiated (2, 4, 6 Gy) and left to form colonies.

### Mitochondrial Iron Measures

Mito-FerroGreen (M489) was used as a fluorescent probe for mitochondrial Fe^2+^. Following treatment, cells were washed with PBS, trypsinized, and pelleted by centrifugation. The cell pellets were washed once with 1X phosphate-buffered saline (PBS) before resuspending with 5 µM Mito-FerroGreen and incubating for 30 min at 37°C. After incubation, the cells were centrifuged, and residual stain was removed from the cell pellet. The cell pellets were washed with PBS 3 times and then resuspended in PBS for analysis. The cells were analyzed using the FITC channel of a Becton Dickinson LSR II flow cytometer.

### DNA Damage Analysis

DNA breaks were detected using alkaline comet assays. The R&D Comet Assay Electrophoresis Starter Kit (#4250-050-ESK) was used following the manufacturer’s instructions. Briefly, cells in agarose suspension were placed on 2-well comet slides and allowed to dry in the dark at 4°C for 10 min. The slides were submerged in lysis buffer for 45 min at 4°C and then incubated for 20 min in alkaline buffer at room temperature. Electrophoresis was carried out at 21 V for 30 min. The slides were then washed twice in water and once in 70% ethanol and were then left to dry at 37°C for 15 min. 1X SYBR Gold (#S11494, ThermoFisher) stain was used to stain the slides for 30 min at room temperature in the dark. The slides were briefly rinsed with water to remove excess stain and were then dried at 37°C for 10 min. Fluorescent microscopy was used to capture images, and analysis was carried out using the autoanalyzer software CometScore 2.0.0.38 to obtain percent tail DNA (http://rexhoover.com/index.php? id=cometscore, access obtained on December 21, 2021).

### Orthotopic Tumor Growth

For all animal studies, methods used were in accordance with standards set by the Institutional Animal Care and Use Committee (IACUC #3111207). Before injection, U251-luciferase cells were trypsinized and the pellet was resuspended in 5% methylcellulose. Female nude athymic (NU/J) mice (Jackson Labs, RRID: IMSR_JAX: 002019) were anesthetized using a ketamine (87.5 mg mL^−1^) xylazine (12.5 mg mL^−1^) cocktail by the University of Iowa IACUC. Following anesthesia, a 1-cm incision was made to expose the skull, and a burr hole was made approximately 3 mm lateral (right) and 2 mm posterior to the bregma. A syringe containing cell +5% methylcellulose was inserted 3 mm deep into the burr hole and 6 × 10^5^ cells in 4 µL were injected over 1 min. Following completion of the surgery, mice received a 5-day treatment of meloxicam (2 mg kg^−1^) for pain management. Tumor growth was monitored by bioluminescence imaging using an Invitrogen IVIS system 10 min following injection of a Luciferin substrate (0.375 mg kg^−1^; Pierce’s D-luciferin, Thermo Fischer Scientific, 88291). Neuroanatomical disruptions were evaluated by collecting a T2 weighted MRI scan 7T Discovery MR901 system (GE Healthcare) using a body transmit coil and a 2-channel mouse brain receiver coil. A 3D FIESTA sequence was applied for anatomical T2-weighted images (in-plane resolution of 104 µm^2^, slice thickness = 160 µm, pixel bandwidth = 326 Hz, flip angle = 30°, TE/TR = 3/6.1 ms, number of averages = 4, and scan time of 9 min and 28 s). Mice were euthanized and counted for overall survival analysis once they met euthanasia requirements per the IACUC protocol, which includes extreme weight loss and hunched posture or tumor growth exceeding the skull.

### Subcutaneous Xenografts

U251 cells were trypsinized and resuspended in sterile saline before subcutaneous injection (5 × 10^6^ cells in 100 µL) into the right rear flank of female NU/J mice (RRID: IMSR_JAX: 002019). Masses were allowed to grow to ∼750 mm^3^ before being treated daily with 10 mg kg^−1^ or an equivalent volume of saline control for 5 days. Tumor volume was calculated as length × (width)^2^/2. Mouse weights were monitored daily. Immediately following the last treatment, mice were euthanized, and tumors were harvested. The tumors were homogenized in collagenase to form a single cell suspension for analysis.

### Statistical Analyses

All statistical analyses were completed with GraphPad Prism software (V.10) with specific statistical tests being noted in the associated figure legend. Synergy calculations were completed with SynergyFinder software (https://synergy­finder.aittokallio.group/20260304125118781437/).

## Results

### Mitochondrial Iron Levels Are Associated with GBM Cell Proliferative Capacity

Because mitochondrial iron sits at the nexus of iron metabolism, we initially evaluated the importance of mitochondrial transport with respect to GBM cell proliferation. Mitoferrin-1 (Mfrn-1) is an iron transporter that facilitates the movement of iron across the inner mitochondrial membrane for utilization.^17^ DepMap analysis of the top 100 codependencies for Mfrn-1 revealed that most codependent genes (37%) are associated with the maintenance of genomic stability and/or DNA damage responses (Figure 1A). Based on this preliminary data analysis, the relationship between Mfrn-1 expression and proliferation in GBM cells (U251, GBM06, and GBM39) was first characterized. Initial observations show that GBM39 cells have significantly higher levels of Mfrn-1 expression which is coupled with significantly lower levels of mitochondrial iron which was evaluated semiquantitatively using Mito-FerroGreen (Figure 1B and C). Moreover, GBM39 cells exhibit significant impairments in colony formation and growth, while the U251 cells, having the highest levels of mitochondrial iron, form robust colonies and have over 2-fold lower doubling time (Figure 1D and E). Taken together, these data suggest that the maintenance of sufficient mitochondrial iron levels may be an important feature of sustained GBM cell proliferation.

**Figure 1.**
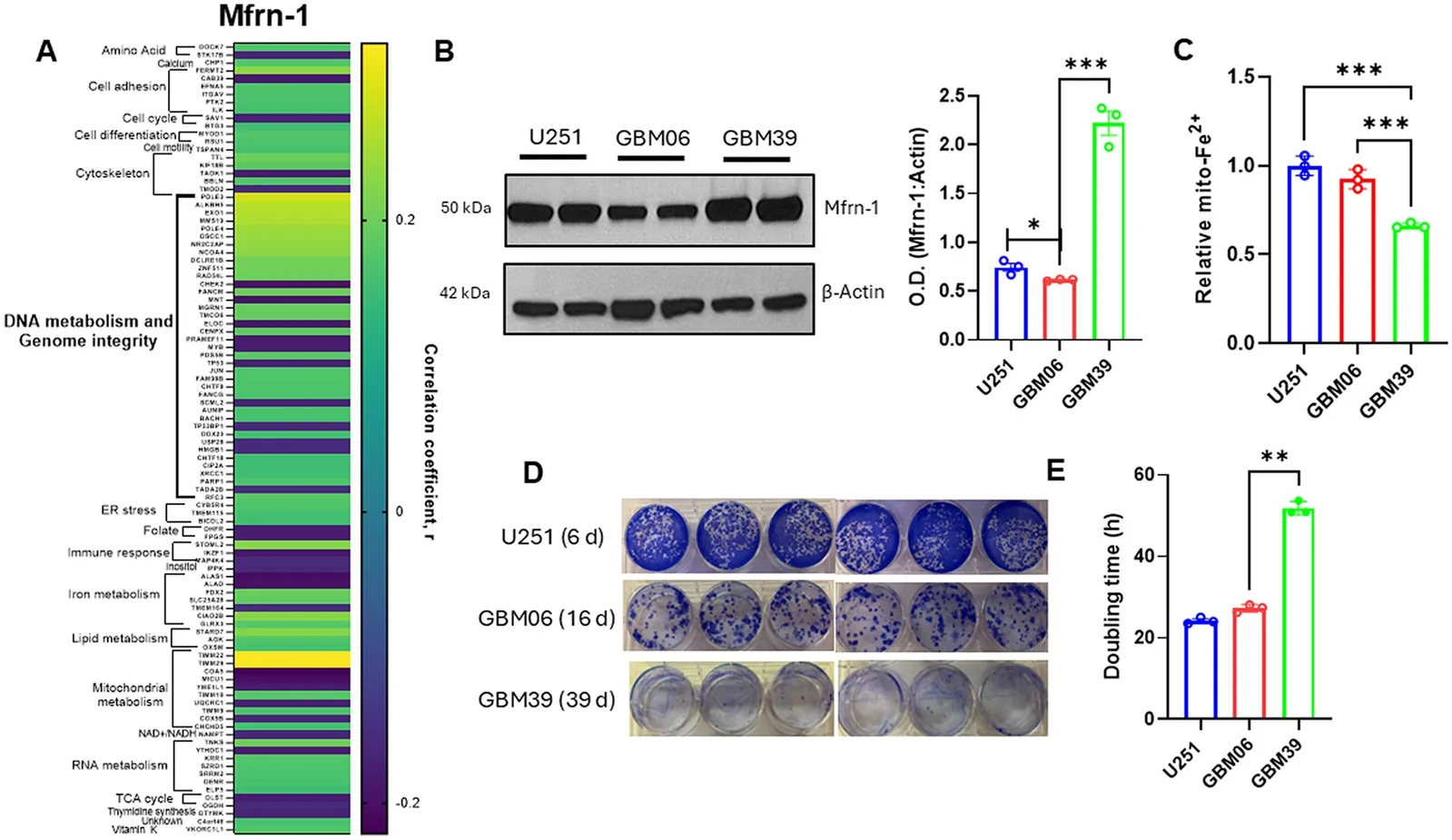
Mitochondrial iron levels are associated with GBM cell proliferative capacity. (A) Top 100 CRISPR codependencies for Mfrn-1 from the DepMap Public 25Q2 database. (B) Western blot analysis of Mfrn-1 expression in U251, GBM06, and GBM39 cell lines where each lane was loaded with 2 technical replicates. This experiment was performed with 3 replicates for quantification where the error bars represent mean ± SEM with *P < .05 and ***P < .0001 using a 1-way ANOVA with a post hoc Tukey’s test for multiple comparisons. (C) Relative mitochondrial iron levels evaluated using Mito-FerroGreen in U251, GBM06, and GBM39 cells. Error bars represent mean ± SEM with ***P < .0001 using a 1-way ANOVA with a post hoc Tukey’s test for multiple comparisons. (D) Colony formation in U251, GBM06, and GBM39 cells. Five hundred individual cells were plated and allowed to grow freely to form colonies. (E) Doubling time over 72 h in U251, GBM06, and GBM39 cells. Error bars represent mean ± SEM with **P < .001 using a 1-way ANOVA with a post hoc Tukey’s test for multiple comparisons. Abbreviations: GBM, glioblastoma; Mfrn-1, Mitoferrin-1.

### Gallium Maltolate Induces DNA Damage

As it appears that the maintenance of mitochondrial iron may be important for tumor cell growth, we aimed to evaluate the effects of GaM as a therapeutic strategy. Gallium can induce mitochondrial iron depletion,^16^ thus, we hypothesized that GaM would promote DNA damage. It was first validated that GaM does, in fact, significantly deplete mitochondrial iron in the most aggressive U251 cells (Figure 2A). The depletion of mitochondrial iron coincided with a significant increase in DNA damage and inhibition of colony formation (Figure 2B and C). As tumor oxygen tension can be variable, we next evaluated the effects of cell culture O_2_ levels on GaM sensitivity. No differences were observed in cells cultured at either 21% or 4% O_2_; however, the induction of γH2AX as a marker of double-stranded DNA damage was more pronounced at 4% (Figure 2D and E). Therefore, it appears that cells with high proliferative rates and increased levels of mitochondrial iron are sensitive to GaM-induced DNA damage.

**Figure 2.**
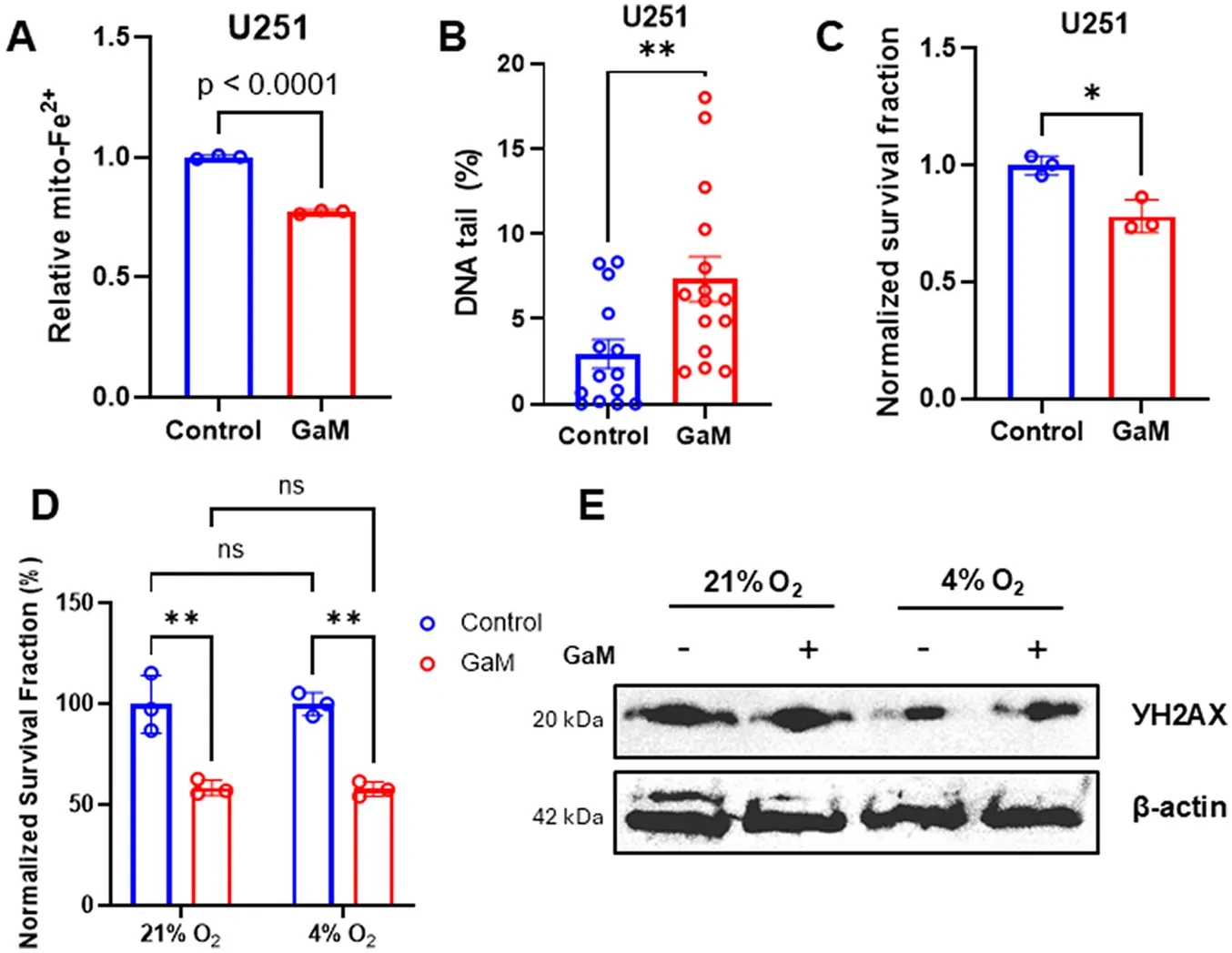
Gallium maltolate depletes mitochondrial iron and induces DNA damage in vitro. (A-C). Mitochondrial iron content measured using Mito-FerroGreen (A), DNA damage induction (B), and clonogenic survival (C) in U251 cells following a 24-h, 50-µM treatment of GaM. Error bars represent mean ± SD where *P < .05. (D, E) Clonogenic survival analysis (D) and γH2AX expression (E) in U251 cells following a 24-h, 50-µM treatment of GaM when cultured at either 21% or 4% O_2_. Error bars represent mean ± SD where *P < .05 and **P < 0.001.

To further assess the ability of GaM to induce DNA damage, U251 cells were implanted in NU/J mice to form subcutaneous xenografts. Once the xenografts formed large masses (≥750 mm^3^), mice were treated with 10 mg kg^−1^ GaM for 5 consecutive days at which point the tumors were harvested for analysis (Figure 3A). Gallium maltolate did not significantly affect mouse weights or tumor volumes throughout treatment (Figure 3B and C). Following harvest, the tumors were mechanically homogenized through a cell strainer to remove the extracellular matrix and generate a single cell suspension. A significant decrease in the number of cells from GaM treated tumors were noted; however, the difference was not significantly lower when normalized to the tumor volume (Figure 3D and E). Consistent with the induction of DNA damage, cells isolated from the GaM-treated tumors showed higher levels of DNA damage evaluated with an alkaline comet assay and phosphorylated γH2AX, indicating that GaM can impact genome stability in vivo as a single agent (Figure 3F and G). Taken together, these data support the hypothesis that GaM is a useful therapeutic strategy to deplete mitochondrial iron and destabilize the genome in highly proliferative GBM cells.

**Figure 3.**
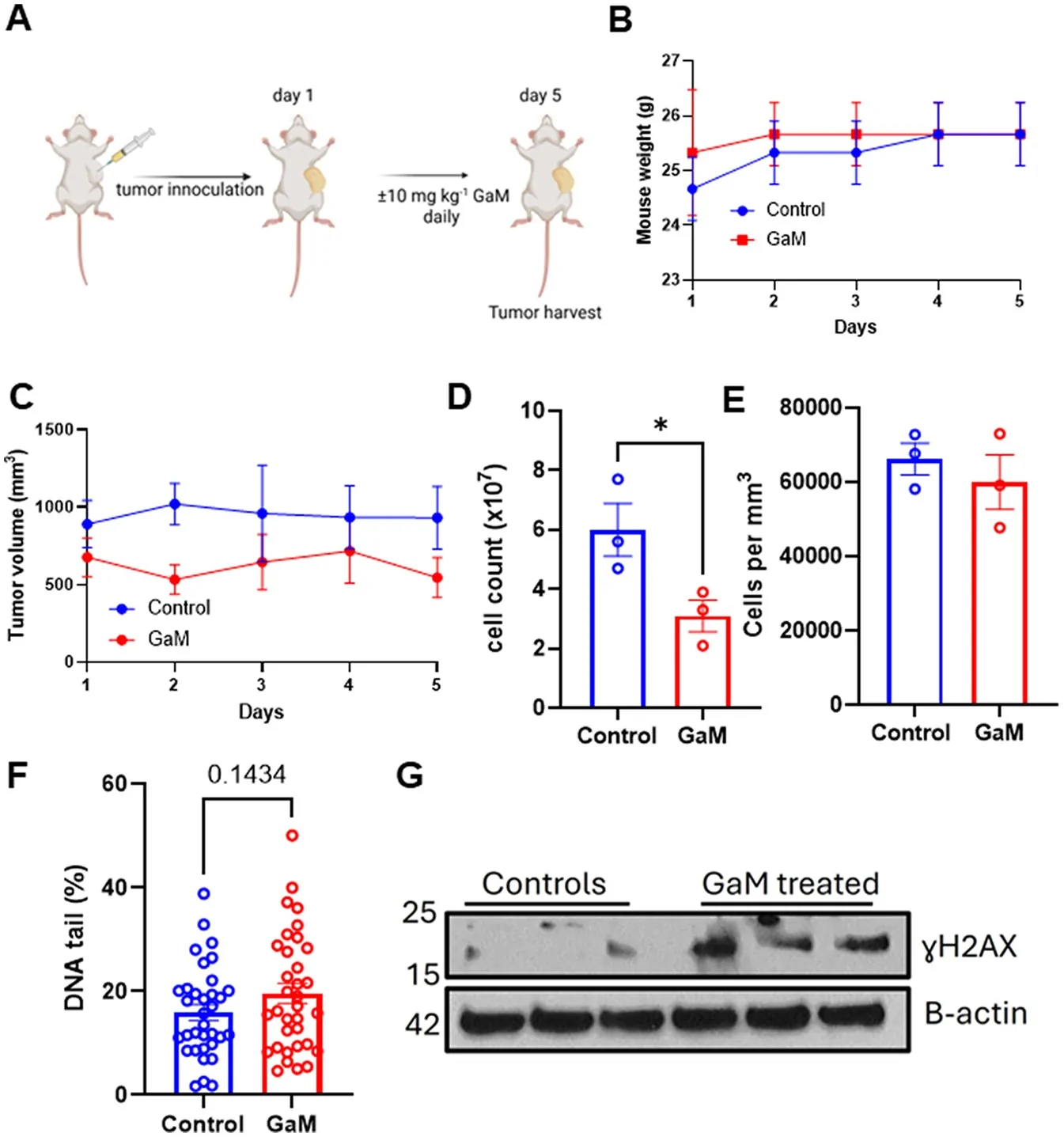
Gallium maltolate induces DNA damage in vivo. (A) Flank xenograft study design where U251 cells were implanted subcutaneously in the rear flank and then treated for 5 consecutive days of GaM (10 mg kg^−1^) intraperitoneally (*n* = 3 per group). After treatment, tumors were harvested and homogenized into a single cell suspension for analysis. (B, C) Mouse weights (B) and tumor volumes (C) throughout the duration of the treatment. (D and E). Direct (D) and volume normalized (E) cells counts from the tumors harvested after treatment. (F, G) DNA damage analysis using an alkaline comet assay (F) and immunoreactive γH2AX levels via western blotting (G) in the homogenized tumor cell suspension harvested from the tumors grown subcutaneously and treated ± GaM (*n *= 3 independent tumors). Abbreviation: GaM, gallium maltolate.

### GaM Synergizes with Ionizing Radiation at Therapeutic Doses

Based on the previous observation that GaM induces DNA damage, it was hypothesized that it can exacerbate radiation-induced DNA damage. To test this hypothesis, U251 cells were treated with 50 µM GaM prior to treatment with 2 Gy irradiation where it was observed that GaM pretreatment sensitized cells to low-dose irradiation and enhanced radiation-induced DNA damage (Figure 4A and B). To further test the utility of GaM as a radiosensitizer, we aimed to evaluate its synergism with ionizing radiation. To test this, U251 cells were preplated as single cells in 6-well dishes to facilitate colony formation as a test of clonogenic survival. Cells were allowed 24 h to attach to the surface of the cell culture dish and then plated with 3 increasing doses of GaM (12.5-50 µM). After a 24-h treatment, the drug was removed and then cells were irradiated with 3 increasing doses of radiation (2-6 Gy, Figure 4C). Cells were given 7 days to form colonies, stained, and counted (Figure 4D). Gallium maltolate alone exhibited significant toxicity at doses >12.5 µM (IC50 ≈ 25 µM; Figure 4E). It was observed that GaM enhanced radiation-induced clonogenic cell killing, but there is only moderate synergy between GaM and radiation at GaM doses above 25 µM (Figure 4F and G). Therefore, GaM appears to have enhanced toxicity in combination with radiation at therapeutic doses.

**Figure 4.**
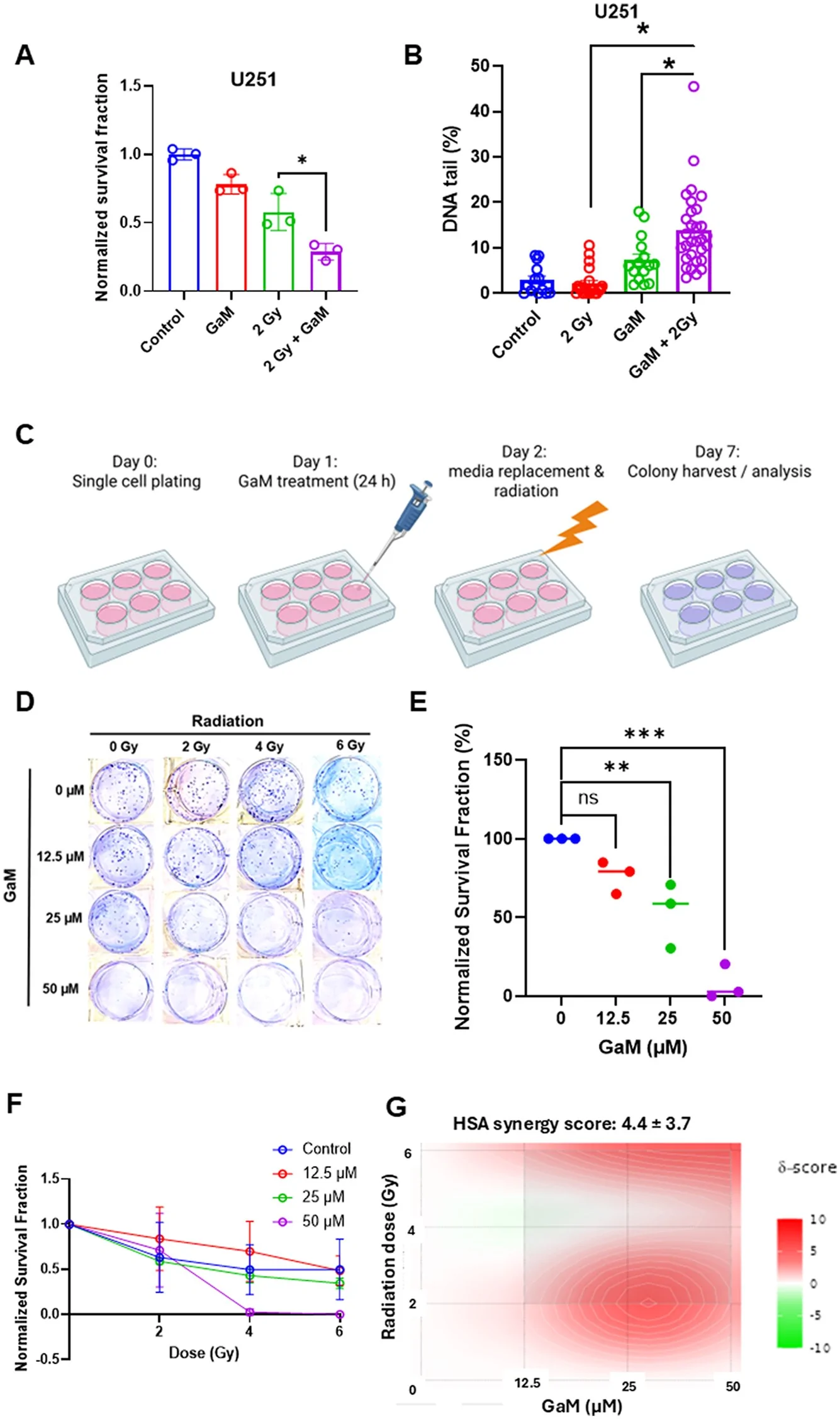
GaM synergizes with ionizing radiation at therapeutic doses. (A, B) Clonogenic survival (A) and alkaline comet assay (B) analysis of U251 cells treated with 50 µM GaM prior to being irradiated with 2 Gy. Error bars represent mean ± SEM (*n* = 3), where *P < .05 using a 1-way ANOVA test with a post hoc Tukey’s test for multiple comparisons. (C, D) Schematic representation of synergy experimental design where cells were initially plated as single cells, given 24 h to attach and treated with increasing doses of GaM (12.5, 25, 50 µM) for 24 h. GaM was removed and plates were exposed to increases doses of ionizing radiation (2, 4, 6 Gy). Cells were given 7 days to form colonies (C). Representative images of the colonies formed following treatment (D). (E) GaM dose response (0-50 µM) in U251 cells analyzed via clonogenic survival. (F) Quantitative analysis of normalized survival fractions to assess the combinatorial effects of GaM and radiation. Analysis was done as a function of radiation dose where clonogenic survival was normalized to the respective GaM-dependent cell killing to control for drug effects. (G) Two-dimensional synergy map generated using SynergyFinder software. Regions in red indicate dose regions with synergy (*δ*) scores >10 indicating synergistic effects. Abbreviation: GaM, gallium maltolate.

### GaM Enhances GBM Response to Radiation and Temozolomide

Following the observed combinatorial effects with ionizing radiation, the effects of GaM on standard of care GBM therapy were tested. Radiation and temozolomide are the current standard of care therapeutic regimen for patients diagnosed with GBM and both therapies are reliant on the induction of DNA damage.^18^ Therefore, we hypothesized that GaM would provide a therapeutic benefit when combined with radiation and temozolomide. Consistent with this hypothesis, GaM treatment 24 h prior to a treatment of 2 Gy and subsequent 1 h of temozolomide led to significant enhanced clonogenic cell killing (Figure 5A and B). To further test the translational potential for this hypothesis, U251 cells were implanted intracranially and treated with GaM in combination with radiation and temozolomide to evaluate in vivo efficacy (Figure 5C). Mouse weights were monitored through the duration of the treatment with GaM, radiation, and temozolomide which revealed no significant changes (Figure 5D). Bioluminescent imaging was employed to monitor tumor growth rates where it was observed that GaM with radiation and temozolomide significantly impaired tumor growth (Figure 5E and F). All treatments were stopped after day 53. On day 93, 3 SOC and 3 SOC+GaM mice were still alive where they were imaged with a T2-weighed MRI to assess potential differences in gross anatomy. At this point, it was observed that there were significant distortions of the lateral ventricles in the SOC treatment mice that were not present in the GaM-treated mice indicating tumor-associated mass effects (Figure 5G). Lastly, mice were monitored for overall survival where it was observed that the addition of GaM to SOC enhanced median overall survival from 127 to 151 days (Figure 5H). As of day 300, 1/5 (20%) SOC and GaM mice were still alive. Taken together, GaM appears to have potential as a useful therapeutic addition to radiation and temozolomide.

**Figure 5.**
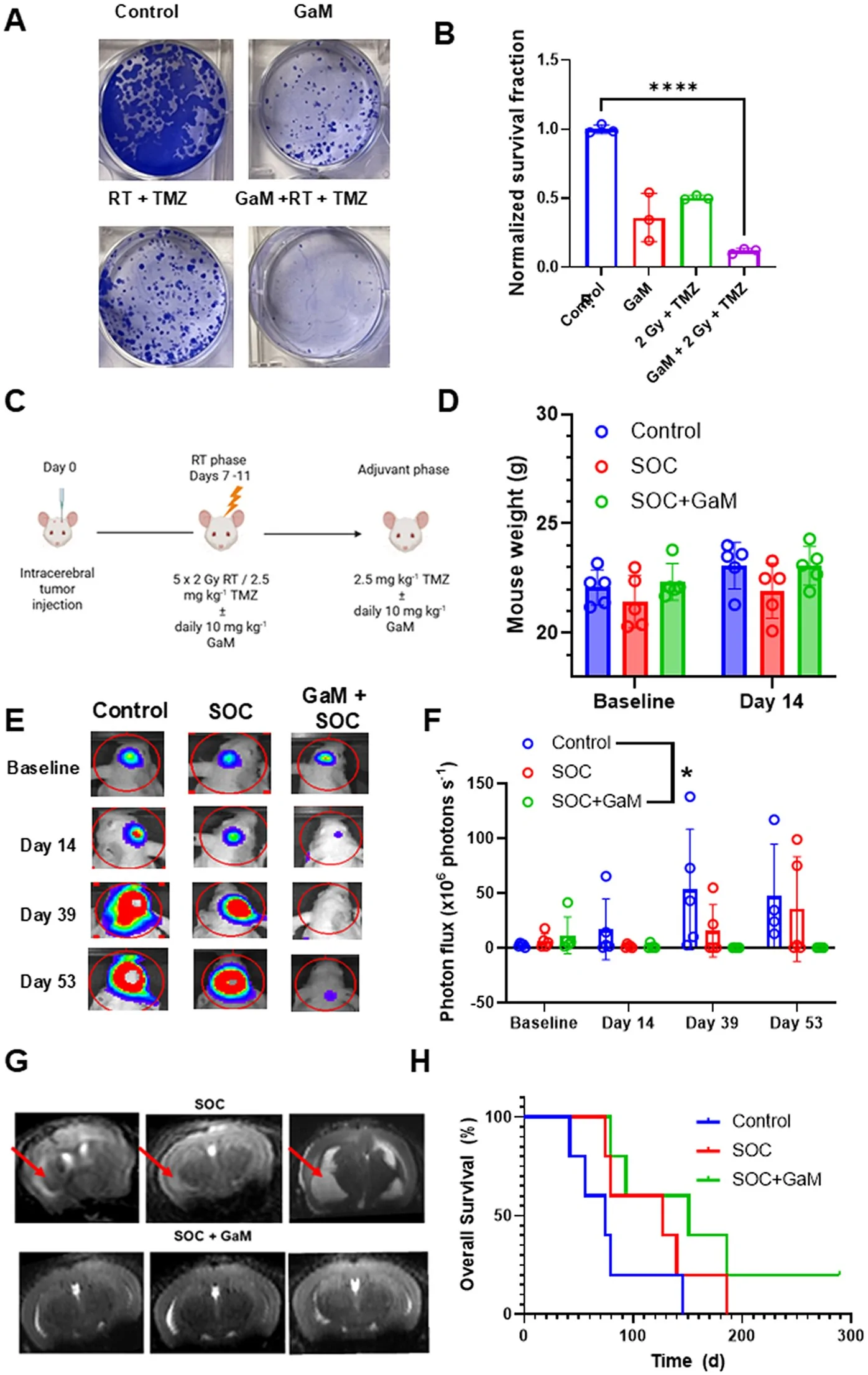
GaM enhances GBM response to radiation and temozolomide. (A, B) Clonogenic survival of U251 cells treated with ± 50 µM GaM for 24 h prior to treatment with 2 Gy irradiation followed by 1 h, 2.5 µM TMZ. Error bars represent mean ± SEM where *P < .05 and **** *P* < 0.00001 using a 1-way ANOVA test with a post hoc Tukey’s test for multiple comparisons. (C) Orthotopic mouse experimental design where mice were implanted with U251 cells transduced with firefly luciferase and confirmed for tumor formation 7 days following implantation. Following confirmation, mice began treatment with standard of care (daily 2 Gy irradiation and weekly 2.5 mg kg^−1^ temozolomide) ± 10 mg kg^−1^ GaM daily. Starting day 14 temozolomide ± GaM were continued adjuvantly until day 53. (D) Change in mouse weight following the completion of the radiotherapy with temozolomide ± GaM. (E, F) Representative images (E) and quantified (F) changes in bioluminescence throughout active treatment. Error bars represent mean ± SEM with *P < .05 using a 2-way ANOVA with a post hoc multiple comparison test. (G) T2-weighted anatomical images at day 93 in the 3 surviving animals from each treatment cohort (SOC and SOC + GaM) showing anatomical disruptions in SOC-treated animals as evidenced by distortions of the lateral ventricles (red arrows). (H) Kaplan-Meier analysis of overall survival from each treatment group (control, SOC, and SOC + GaM). Abbreviations: GaM, gallium maltolate; GBM, glioblastoma; TMZ, temozolomide.

## Discussion

Mitochondrial iron trafficking plays a central role in the maintenance of iron metabolism as it houses both iron-sulfur cluster biogenesis and heme synthesis machinery.^19^ Importantly, mitochondrial iron serves as a central connection point between iron and DNA metabolism because nearly all major DNA metabolic enzymes require a completed iron-sulfur cluster in order to carry out their functions in performing high fidelity DNA replication and maintaining genomic stability.^13^ Hence, inhibition of iron-sulfur cluster biogenesis has been theorized to be an important therapeutic target for aggressive tumor cells.^12^ This principle is further supported by our initial bioinformatic data showcasing that most Mfrn-1 genetic codependences are related to DNA replication and DNA damage repair. These bioinformatic results are congruent to our initial in vitro assessment that GBM cells with endogenously high Mfrn-1 expression are significantly less proliferative. The Mfrn-1 high expressing cells had significantly lower levels of mitochondrial iron which would suggest that the elevated expression is an adaptive response that is associated with an endogenous iron limitation that has yet to be robustly explored. While it is currently unclear what unique characteristics drive this iron limited phenotype, it has been established in erythroid cells that limiting mitochondrial iron will induce an adaptive elevation of Mfrn-1.^20^ Taken together, it can be ascertained that mitochondrial iron is an important substrate to support the enhanced proliferation of aggressive GBM tumor cells.

Given the potential importance of mitochondrial iron in maintaining genome stability to support GBM cell proliferation, strategies to impair its trafficking would be paramount and have potential to strategically target highly proliferative GBM cells that may lead to disease progression. Gallium maltolate represents a promising therapeutic to achieve this goal. Mechanistically, GaM can induce DNA damage in GBM cells and enhance the efficacy of DNA damaging agents ionizing radiation and temozolomide in vitro. To this point, gallium-based therapies have been most closely associated with the inhibition of ribonucleotide reductase or the electron transport chain.^15^ It was recently shown that Ga(NO_3_)_3_ can deplete mitochondrial iron to induce DNA damage^16^; however, this is the first study to demonstrate that GaM can be translationally relevant by facilitating the same mechanism. Consistent with the overarching premise that enriched mitochondrial iron content can provide a necessary substrate for tumor proliferation, it has been reported that Mfrn-1 overexpression can drive GBM cell proliferation, colony formation, and orthotopic tumor growth suggesting that mitochondrial iron can be a promoter of proliferation as opposed to a consequence of increased proliferation.^21^ However, this effect was not tested in relation to genomic stability. Therefore, the full impacts of mitochondrial iron trafficking need to be more robustly interrogated from a fundamental biological perspective. Taken together, we propose that GaM toxicity occurs through the depletion of mitochondrial iron in highly proliferative GBM cells that are reliant on mitochondrial iron for the maintenance of genomic stability to support enriched growth (Figure 6). This is ideal from a therapeutic standpoint because proliferative cells are more likely to contribute to disease progression and first-line treatment failures.

**Figure 6.**
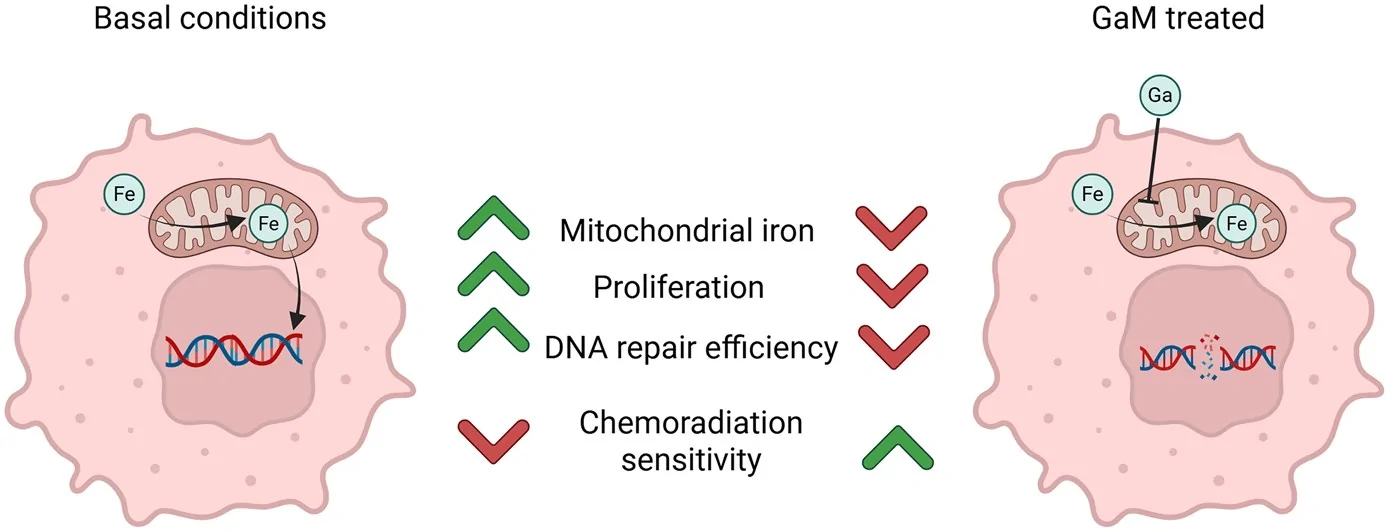
Working model. Based on the results presented in this study it can be hypothesized that efficient mitochondrial iron trafficking helps to support the rapid proliferation of aggressive GBM tumors by helping to facilitate effective DNA repair to meet the high proliferative demands. In this context, gallium maltolate can serve as a useful therapeutic strategy to impair mitochondrial iron trafficking and promote genomic instability in highly proliferative cells. This results in enhanced sensitivity to DNA damaging agents such as radiotherapy and temozolomide. Abbreviation: GBM, glioblastoma.

From a translational science viewpoint, GaM has gained considerable interest within the neuro-oncology community, as it has previously been shown to inhibit GBM tumor growth when treated as a single agent in preclinical models.^22^ This would be consistent with our results demonstrating that GaM alone can induce DNA damage in subcutaneous flank tumor cells, a result that has been previously been reported in vitro using Ga(NO_3_)_3_ but has yet to be shown in an in vivo setting.^16^ Importantly, we have shown that GaM may be more efficacious when given in combination with DNA damaging agents (eg ionizing radiation and temozolomide) both in vitro and in vivo. In an orthotopic model system, GaM with radiation and temozolomide was able to extend median overall survival from 127 to 151 days. Currently, there is no universally accepted survival threshold that directly translates from mice to humans to facilitate a prediction for human survival in preclinical cancer models. However, for context, the addition of temozolomide to conventional radiotherapy in an orthotopic GBM model increased overall survival by 11 days.^23^ Furthermore, a recently completed phase 1 study of GaM in refractory GBM showed a median overall survival of 16 months in 24 subjects where overall survival is only 9 months for patients who receive secondary surgeries and patient survival rarely exceeds 1 year.^24-26^ Taken together, these results suggest that there may be some therapeutic benefit for combining GaM with standard of care that warrants further investigation.

## Conclusions

This study highlights the therapeutic potential for disrupting mitochondrial iron metabolism to enhance GBM outcomes using GaM. It appears that the maintenance of adequate mitochondrial iron content can support tumor cell proliferation and depletion with GaM can induce genome instability both in vitro and in vivo. The apparent genomic instability caused by GaM allows it to synergize with ionizing radiation and enhance the efficacy of standard of care therapy (radiotherapy and temozolomide). These preclinical observations support consideration of GaM for further investigation as an addition to standard GBM therapies.

## Data Availability

The data are available upon request to the corresponding author.
